# Proliferation of Colorectal Cancer Is Promoted by Two Signaling Transduction Expression Patterns: ErbB2/ErbB3/AKT and MET/ErbB3/MAPK

**DOI:** 10.1371/journal.pone.0078086

**Published:** 2013-10-30

**Authors:** Yong-Liang Yao, Jie Shao, Chunfu Zhang, Jian-Hong Wu, Qing-Hui Zhang, Jian-Jun Wang, Wei Zhu

**Affiliations:** 1 Department of Clinical Laboratory, Kunshan First People’s Hospital, Affiliated to Jiangsu University, Kunshan, Jiangsu, China; 2 School of Medical Science and Laboratory Medicine, Jiangsu University, Zhenjiang, Jiangsu, China; 3 Department of Clinical Laboratory, The 359th Hospital of PLA, Zhengjiang, Jiangsu, China; 4 Department of Clinical Laboratory, The Second People’s Hospital of Kunshan, Kunshan, Jiangsu, China; H.Lee Moffitt Cancer Center & Research Institute, United States of America

## Abstract

One of the recent breakthroughs in cancer research is the identification of activating mutations in various receptor tyrosine kinase(RTK) pathways in many cancers including colorectal cancer(CRC). We hypothesize that, alternative to mutations, overexpression of various oncogenic RTKs may also underpin CRC pathogenesis, and different RTK may couple with distinct downstream signaling pathways in different subtypes of human CRC. By immunohistochemistry, we show here that RTK members ErbB2, ErbB3 and c-Met were in deed differentially overexpressed in colorectal cancer patient samples leading to constitutive activation of RTK signaling pathways. Using ErbB2 specific inhibitor Lapatinib and c-Met specific inhibitor PHA-665752, we further demonstrated that this constitutive activation of RTK signaling is necessary for the survival of colorectal cancer cells. Furthermore, we show that RTK overexpression pattern dictates the use of downstream AKT and/or MAPK pathways. Our data are important additions to current oncogenic mutation models, and further explain the clinical variation in therapeutic responses of colorectal cancer. Our findings advocate for more personalized therapy tailored to individual patients based on their type of RTK expression in addition to their mutation status.

## Introduction

Colorectal cancer is an epithelial malignancy occurring in the colon or rectum. More than 1 million people are diagnosed with colorectal cancer yearly worldwide resulting in about 0.5 million deaths. In 2008, it was the second most common cause of tumor-associated death in women and the third most common in men. It is more common in developed than in developing countries [1]–[3]. Chemotherapy may be used as adjuvant therapy in addition to surgery in almost all colorectal cancer patients [4]–[6]. With or without lymph node metastasis, chemotherapy is considered to increase life expectancy and reduce the possibility of reoccurrence. Chemotherapy drugs are designed to act via a variety of mechanisms and may include combinations of agents such as fluorouracil, capecitabine, UFT, leucovorin, irinotecan, or oxaliplatin [7]–[9]. However, for various reasons, chemotherapy can cause other problems for the patients mainly due to its non-specific attack and related side effects, such as nausea, vomiting, hair loss, fatigue, anemia and infection among others. Therefore, targeted cancer therapy has emerged which has been developed based on knowledge of cell signaling. Targeted cancer therapy mainly depends on small-molecule drugs and monoclonal antibodies which cause far fewer side effects compared to traditional chemotherapy.

Exogenous signals such as epidermal growth factor (EGF) and hepatocyte growth factor (HGF) were previously reported to be vital to maintain sustained growth of tumor cells by binding to their receptors. These receptors belong to the receptor tyrosine kinases (RTKs) family and ideal targets in cancer therapy as demonstrated in many studies. After systemic profiling of cancer genomes by using the next generation sequencing, we now know that acquisition of activating mutations in RTKs or downstream signaling molecules is one of the major oncogenic mechanisms. However, only a small subset of patients possess these mutations, suggesting that other alternative mechanisms are at play. One of these alternative mechanisms could be the overexpression of pro-proliferation and pro-survival RTKs, and/or differential usage of downstream signaling pathways. With more in-depth analysis of cancer genomes, we are now begin to understand the individual differences which signify unique disease biology and clinical management requirement.

The family of epidermal growth factor receptor (EGFR) and c-Met are RTKs that have been frequently reported in colorectal carcinoma progression and metastasis. Activation of these RTKs can stimulate a number of specific pathways directly effecting tumor cell migration, survival and proliferation. The aberrant regulation of the RTKs is often noted in advanced CRC. Compared to EGFR, other EGFR family members, c-MET, ErbB2, ErbB3,and their related signaling pathways are relatively unknow [10]. Therefore, in the present study, we investigated the differential expression of RTKs c-MET, ErbB2, ErbB3, and down stream signaling molecules in primary samples of colorectal cancer patients, as well as in representative human colorectal cancer cell lines.

## Materials and Methods

### Patients

A total of 105 colorectal cancer specimens were investigated in this study and were obtained from patients at the time of surgical resection and endoscopy. Normal colorectal tissues were obtained from 40 Chinese patients with non-tumoral diseases such as tediously long colorectal and vascular malformation. All of the cases were treated at the First People’s Hospital of Kunshan, China between January 2010 and August 2011, and all the samples were collected according to the principles expressed in the Declaration of Helsinki. Written documented informed consent for gene expression analyses of all tissues was obtained from all patients prior to surgery or endoscopy examination. This study and the consent procedure were approved by the local ethics committee of the First People’s Hospital of Kunshan, China. The diagnosis and staging of colorectal cancer was assessed according to the AJCC (TNM) Staging System.

### Cell Culture and Reagents

LoVo, SW948 and SW480 cell lines were obtained from ATCC. EGF was from Sigma (Milan, Italy), HRG1-β1 and IGF-1 from R&D Systems, (Minneapolis, MN). LY294002 was from Calbiochem, U0126 from Promega, PHA-665752 from Tocris Bioscience, and Gefitinib from Sequoia Research Products. All cell lines were cultured in DMEM supplemented with 10% FBS, penicillin (100 U/mL) and streptomycin(100 µg/mL) at 37°C in 5% CO_2_.

### Western Blotting

Proteins were extracted from human tissues and cell lines, and quantitated using a protein assay (Bio-Rad Laboratories, Hercules, CA). Protein samples (30 µg) were fractionated by SDS-PAGE and transferred to a nitrocellulose membrane. Immunoblotting was carried out using antibodies against ErbB2, ErbB3, p-C-MET, C-MET, p-MAPK, MAPK, p-AKT and AKT (Santa Cruz Biotechnology, Inc., Santa Cruz, CA). The results were visualized using a chemiluminescent detection system (Pierce ECL substrate western blot detection system, Thermo Scientific, Rockford, IL) and exposure to autoradiography film (Kodak XAR film).

### Immunohistochemistry (IHC)

All tissues were removed and fixed in 4% paraformaldehyde overnight at 4°C, then processed and sectioned at 5 µm thickness. Sectioned slides were stained immunohistochemically for ErbB2, ErbB3 and c-MET (Santa Cruz Biotechnology, Inc.) followed by incubation with secondary antibody at 37°C for 30 min and reaction with DAB reagent for 5–10 min. The slides were mounted with neutral gum for microscopic examination. Cells with brown intracellular granules (cytoplasm or nucleus) were considered to be positively stained.

### Cell Viability

Cultured cells were plated at a density of 6×10^3^ cells/well in a 96-well plate and treated with various agents. Cell viability was evaluated by an MTS assay. CellTiter 96® AQueous One Solution Reagent (Promega, Madison, WI) was added to each well according to the manufacturer’s instructions, and the plates were returned to the incubator. After 4 hours, cell viability was determined by measuring the absorbance at 490 nm using a computer controlled plate-reader.

### Statistical Analysis

Data are expressed as mean ± standard deviation (SD). Comparisons between two groups were performed using Student’s t-test or the Mann-Whitney U test, as appropriate. All statistical analyses were performed using SPSS statistical software (version 13.0), and two-tailed t-tests were applied to all data unless otherwise specified. A value of *P* less than 0.05 was considered to be statistically significant.

## Results

### Differential Expression of ErbB3, ErbB2 and c-MET in Human Colorectal Cancer

**Figure 1:**
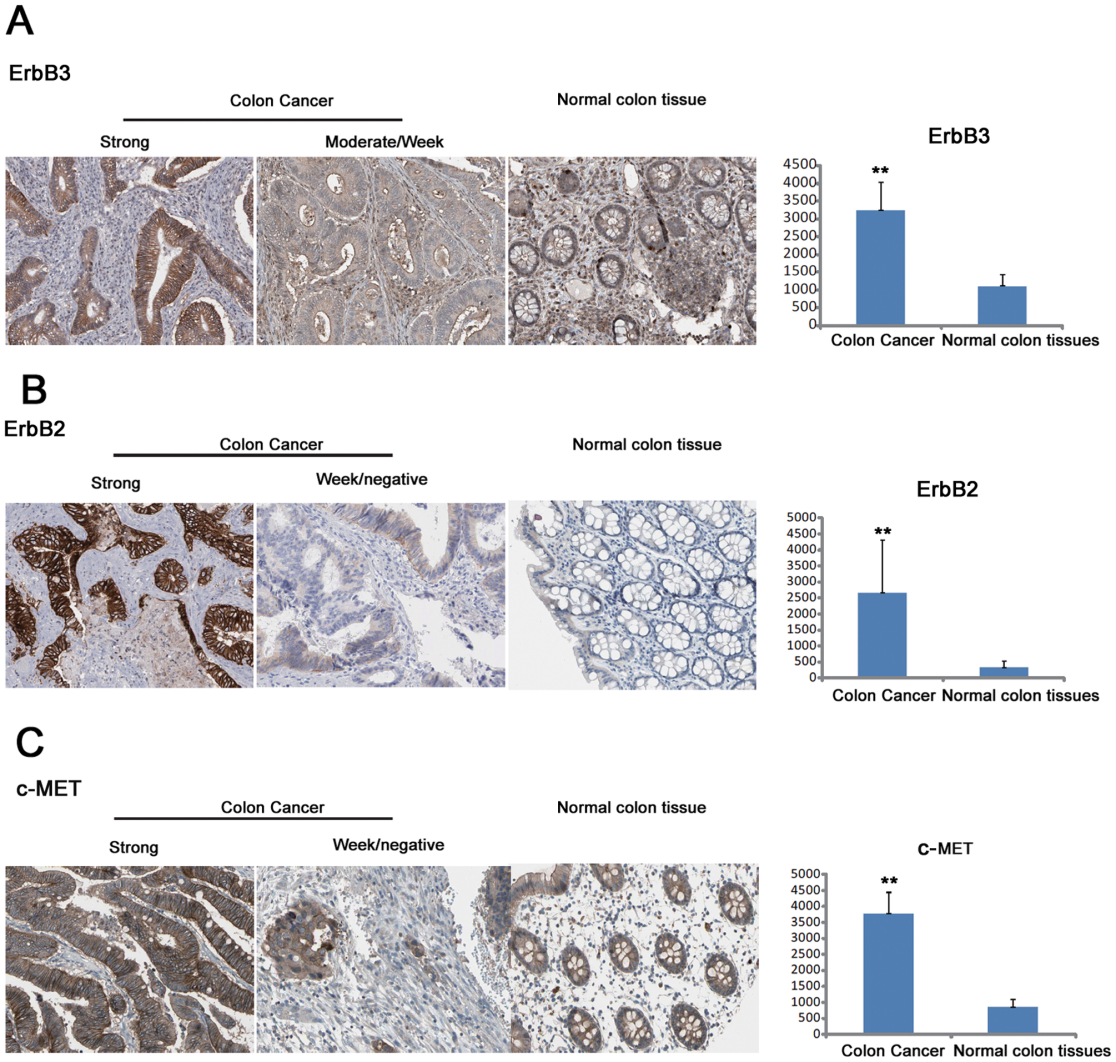
Expression of ErbB3, ErbB2 and c-MET in human colorectal cancer. (A, B, C) Expression of ErbB3, ErbB2 and c-MET in human colorectal cancer. Specimens of 105 paraffin-embedded colorectal cancer tissues were analyzed by IHC for ErbB3, ErbB2 and c-MET, and representative figures show strong and weak/negative staining for each marker with respective normal control samples. Average integrated optical density (IOD) was obtained by analyzing five fields for each slide, evaluated by Image-Pro Plus software (version 5.0) for IHC staining of ErbB3, ErbB2 and c-MET. The data are presented as mean ± SD, and *P<0.05, **P<0.01 by Mann-Whitney U test.

In order to investigate the expression of a set of receptor tyrosine kinase genes (RTK) in human colorectal cancer, paraffin embedded blocks were collected which contained specimens of 105 cases of colorectal cancer. First, the expression of the important oncogenic heterodimer ErbB2/ErbB3 was determined by IHC. As shown in **Figure 1A** **,** ErbB3 was significantly overexpressed in human colorectal cancer tissue, with expression mainly distributed in the membrane of cancer cells. The partner of ErbB3 in the heterodimer, ErbB2, was also located mainly in the membrane of cancer cells and was similarly overexpressed in human colorectal cancer (**Figure 1B**). Another receptor tyrosine kinase, the proto-oncogene C-MET, was also detected by IHC, in the membrane of cancer cells in most cases (**Figure 1C**). Finally, quantitative analysis of expression of the three proteins was performed using the image analysis software Image-pro plus. The IHC-positive areas from five random fields of each IHC-stained section were evaluated as integrated optical density (IOD). All three proteins were significantly overexpressed in human colorectal tissues. (ErbB3: cancer tissues 3264±1032 vs. normal colorectal tissues 1038±354, *P = *0.0074, ErbB2: cancer tissues 2616±2019 vs. normal colorectal tissues 338±154, *P* = 0.016, c-MET: cancer tissues 3812±739 vs. normal colorectal tissues 978±313, *P* = 0.0057 by Mann-Whitney U test).

**Figure 2:**
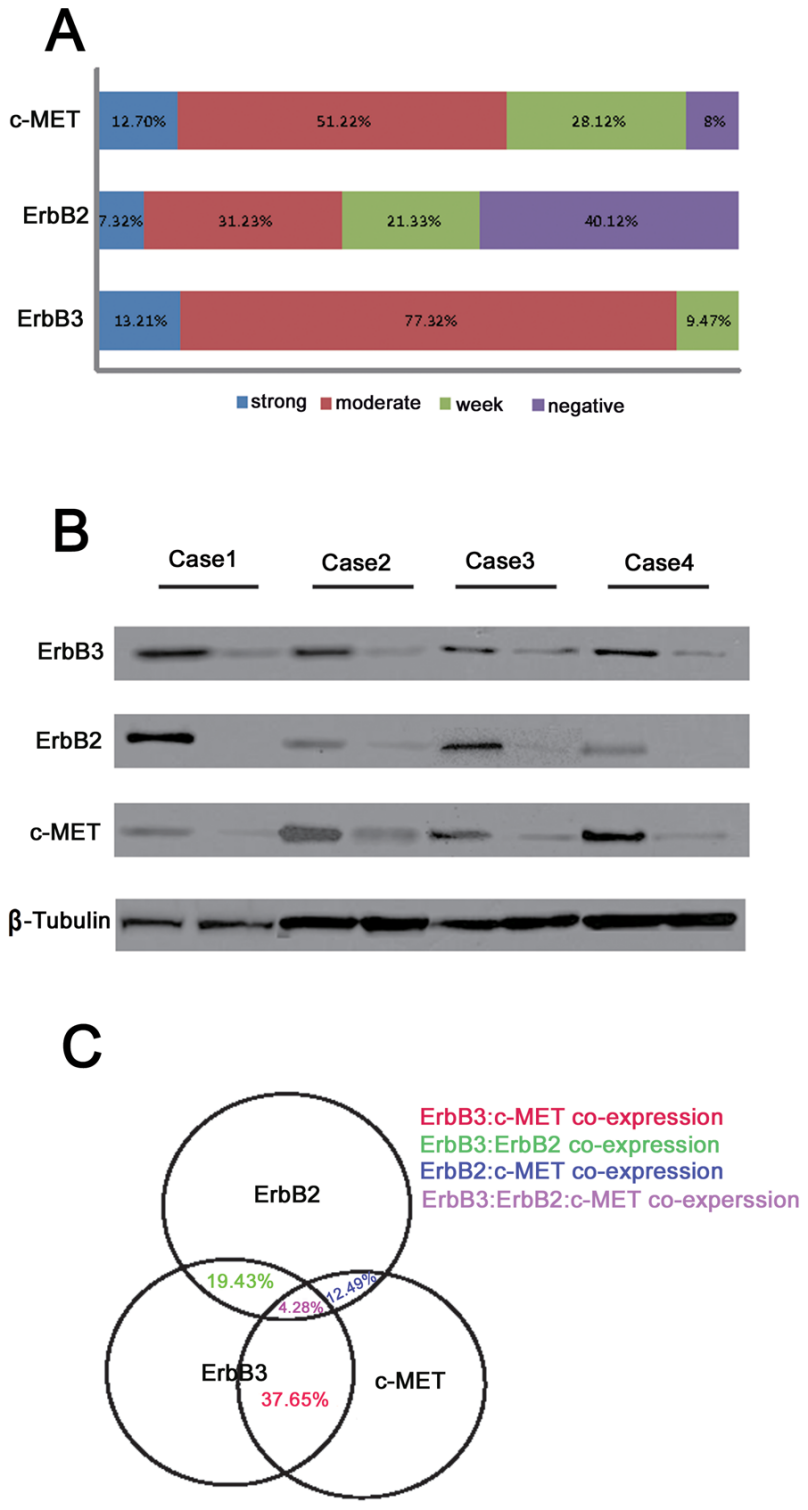
Heterogeneous expression of ErbB3, ErbB2 and c-MET in human colorectal cancer. (A) Ratio of strong/moderate/weak/negative staining of ErbB3, ErbB2 and c-MET in human colorectal cancer. (B) Detection of ErbB3, ErbB2 and c-MET in protein extracted from tissue samples of 4 cases of colorectal cancer. (C) Venn diagram showing co-expression of ErbB3, ErbB2 and c-MET.

The results of expression of all three proteins were expected, not only due to their role as proto-oncogenes, but also because previous similar studies have indicated similar results. However, after further investigation, we found varying degrees of variation in the expression of these three proteins in human colorectal cancer, especially in ErbB2 expression. Of the 105 cases examined, 45 cases (42.85%) were negative for ErbB2 staining, 8 cases showed strong ErbB2 staining, and the remainder (52 cases) showed moderate or weak ErbB2 staining. Only 9 cases (8.5%) were negative for c-MET staining while 14 cases exhibited strong staining and 82 cases showed moderate or weak c-MET staining. Interestingly, ErbB3 was positive in all colorectal cancer tissues examined (**Figure 2A**). Moreover, the heterogeneous expression of these three proteins in four colorectal cancer tissues was further investigated by western-blot (**Figure 2B**). The difference in expression between cancer tissues and normal tissues of all three proteins confirmed the reliability of IHC staining, and further indicated that three types of expression patterns existed in the 105 cases. All the cases examined could be classified as: ErbB3: c-MET co-over-expression colorectal cancer, ErbB3:ErbB2 co-over-expression colorectal cancer, and ErbB3:ErbB2: c-MET co-over-expression colorectal cancer (**Figure 2C**). We believe that these results are a good example of variation of tumor RTK expression pattern, and furthermore, that the molecular mechanisms underlying the different patterns are worthy of exploration.

### Proliferation of Colorectal Cancer Cells is Mediated by Either ErbB3/ErbB2 or ErbB3/C-MET Signaling Pathways

**Figure 3:**
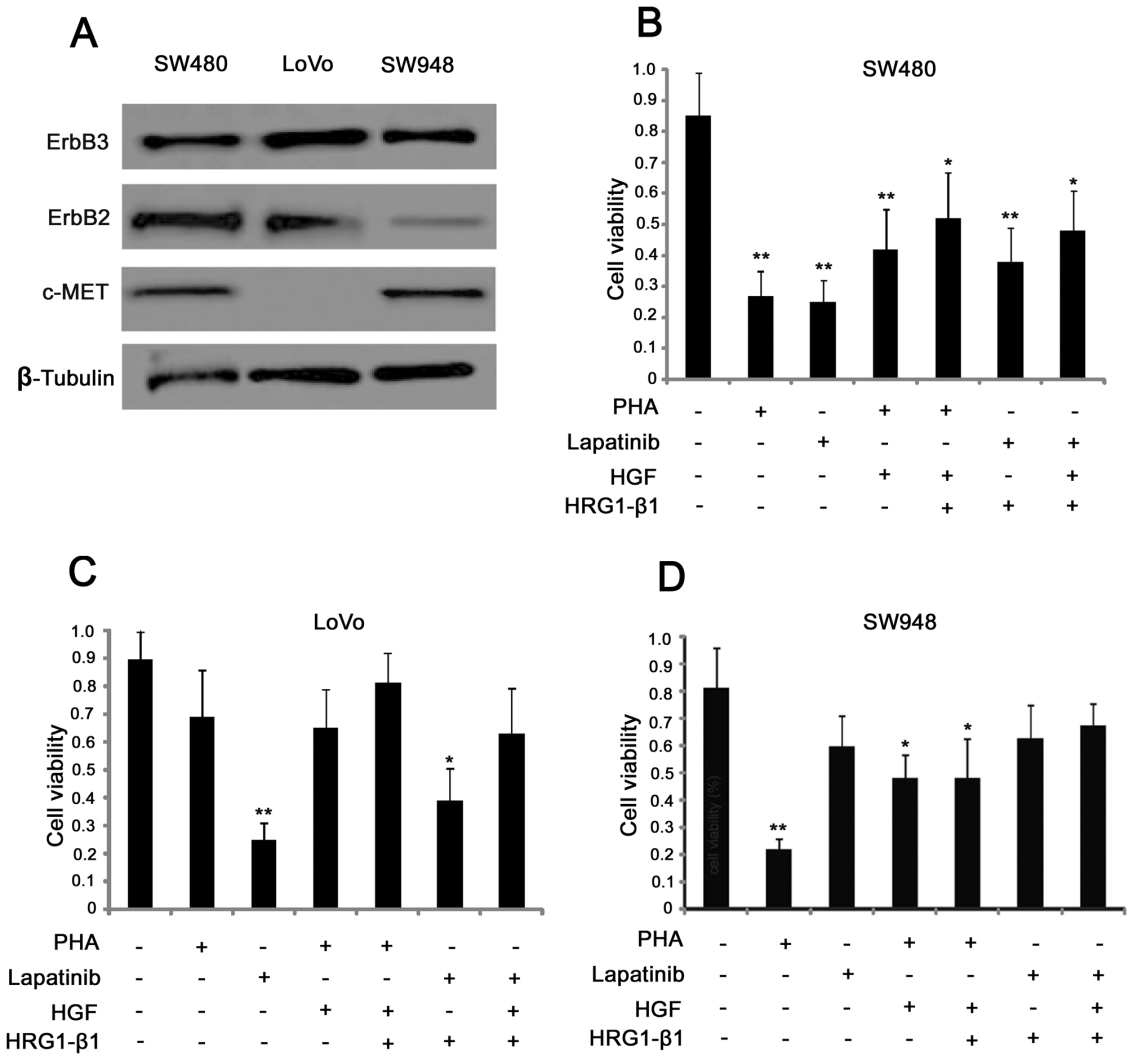
Proliferation of colorectal cancer cells is mediated by both ErbB3/ErbB2 and ErbB3/c-MET signaling Pathways. (A) Detection of ErbB3, ErbB2 and c-MET in the human colorectal cancer cell lines LoVo, sw948 and sw480 by western-blot. (B, C, D) Cell viability measured by MTS assay in the colorectal cancer cell lines LoVo, sw948 and sw480 treated as indicated in the figure. The data are presented as mean ± SD, and *P<0.05, **P<0.01 by unpaired Student’s t test.

In order to identify suitable human colorectal cancer cell lines corresponding to the three molecular patterns which we discovered in the human samples, the expression of ErbB2, ErbB3 and c-MET in 9 human colorectal cancer cell lines was analyzed by western blotting (data not shown). The result showed that SW480 is a representative cell line co-expressing ErbB2, ErbB3 and c-MET. The cell line LoVo only co-over-expresses ErbB2 and ErbB3, while SW948 is a representative cell line which co-over-expresses ErbB3 and c-MET (**Figure 3A**). Cell viability was accessed by MTS assay, and the related mechanisms will be further discussed. Some inhibitors and agonists of these three proteins were selected including PHA-665752 (PHA) and lapatinib, which are inhibitors of c-MET and epidermal growth factor receptor (EGFR), respectively. Once c-MET signaling was blocked by PHA, the cell viability of SW480 and SW948 decreased significantly **(** **Figure 3** **B and D);** however no significant change in cell viability occurred in LoVo cells, perhaps due to their low expression of c-MET **(** **Figure 3C** **)**. Conversely, lapatinib, an ErbB2 inhibitor, significantly reduced the cell viability of LoVo and SW480 cells, while having almost no effect on SW948 cells which have low ErbB2 expression **(** **Figure 3D** **)**. The differences between LoVo and SW948 cells in their response to PHA and lapatinib are possibly due to differences in their signal transduction pattern **(** **Figure 3** **C and D)**. Moreover, in order to investigate whether the agonists can rescue cell viability, HGF, an agonist of c-MET, and heregulin-β1 (HRG1-β1), which binds to HER3 and induces its heterodimerization with the other family members, were used. Both HGF and HRG1-β could partially rescue the viability of SW480 cells **(** **Figure 3B** **)**; however, the situation was different in LoVo and SW948 cells. HGF stimulation caused an insignificant increase of cell viability in LoVo cells compared with LoVo cells treated with PHA **(** **Figure 3C** **)**. SW948 cells responded more sensitively to HGF, with a significant increase in cell viability compared to PHA-treated SW480 cells **(** **Figure 3D** **)**. However SW948 cells responded less sensitively to HRG1-β1 compared to LoVo, perhaps due to their low expression of ErbB2, but the cell viability of SW948 was still increased, possibly due to the other receptors of the EGFR family such as EGFR and ErbB4 which can both dimerize with ErbB3. Therefore, different responses were explored due to different molecular patterns of expression: ErbB3/ErbB2 and ErbB3/c-MET. The down-stream signaling was then investigated further.

### Different Molecular Patterns of Colorectal Cancer Involve the Activation of Downstream MAPK or AKT Signaling

**Figure 4:**
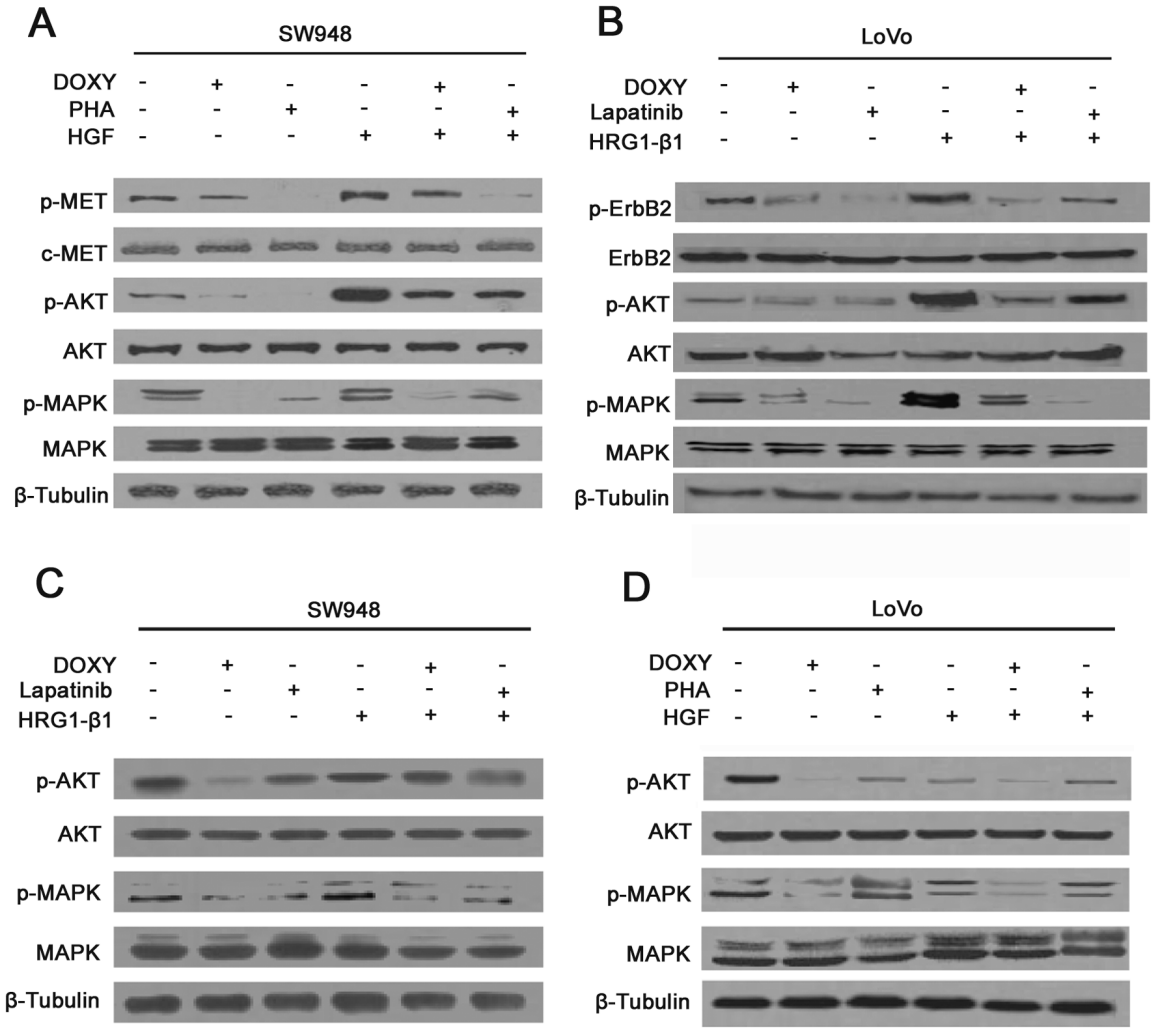
Activation of MAPK or AKT signaling in colorectal cancer cells with different molecular patterns of expression. (A, B) Detection of AKT and MAPK signaling in SW948 and LoVo cells by western-blot, with the treatment indicated in the figure. (C, D) Detection of AKT and MAPK signaling by western-blot in LoVo and SW948 cells with HGF or HRG1-β1 treatment, with or without different agonists or antagonists, as indicated.

In order to investigate further the mechanism of downstream signaling of both molecular patterns in colorectal cancer, cell lysates were utilized to analyze the activation of AKT and MAPK signaling which are well-known downstream signaling pathways of ErbB2/ErbB3 and ErbB3/c-MET complexes. An inducible siRNA system was involved in the study, and the expression of ErbB3 could be silenced by doxycycline (DOXY) treatment. Once SW948 was treated with PHA or DOXY, activation of both AKT and MAPK were blocked. Both these two signaling pathways could be reactivated by HGF, but no significant reactivation was found when ErbB3 was silenced even with HGF treatment, indicating that activation of MAPK signaling in SW948 depended more on ErbB3 **(** **Figure 4A** **).** The activation of AKT and MAPK signaling was accessed in a different way in LoVo cells due to their high expression of ErbB2/ErbB3. Both AKT and MAPK signaling were effectively blocked when treated with DOXY and lapatinib, indicating impairment of both ErbB3 and other EGFR molecules which can inactivate AKT and MAPK signaling. HRG1-β1 was able to strongly activate both AKT and MAPK signaling; however its activation effect on AKT was more dependent on ErbB3 while its activation effect on MAPK was more dependent on other EGFR family members **(** **Figure 4B** **)**. Furthermore, the treatment conditions are reversed between these two cells. Lapatinib has little effect on activation in SW948 cells, but HRG1-β1 can still induce activation of both AKT and MAPK signaling although the activation degree is much weaker than in LoVo cells **(** **Figure 4C** **)**. Similarly, the c-MET inhibitor PHA alone has almost no effect on AKT and MAPK signaling, and HGF can barely activate either AKT or MAPK signaling probably due to its low expression of c-MET **(** **Figure 4D** **)**. The specificity of the growth signaling via ErbB3/ErbB2 or ErbB3/c-MET was further studied using specific inhibitors.

### AKT and MAPK are Two Cell-signaling Pathways Governing Cell Viability of Colorectal Cancer Cell Lines with Different Receptors Expression Patterns

**Figure 5:**
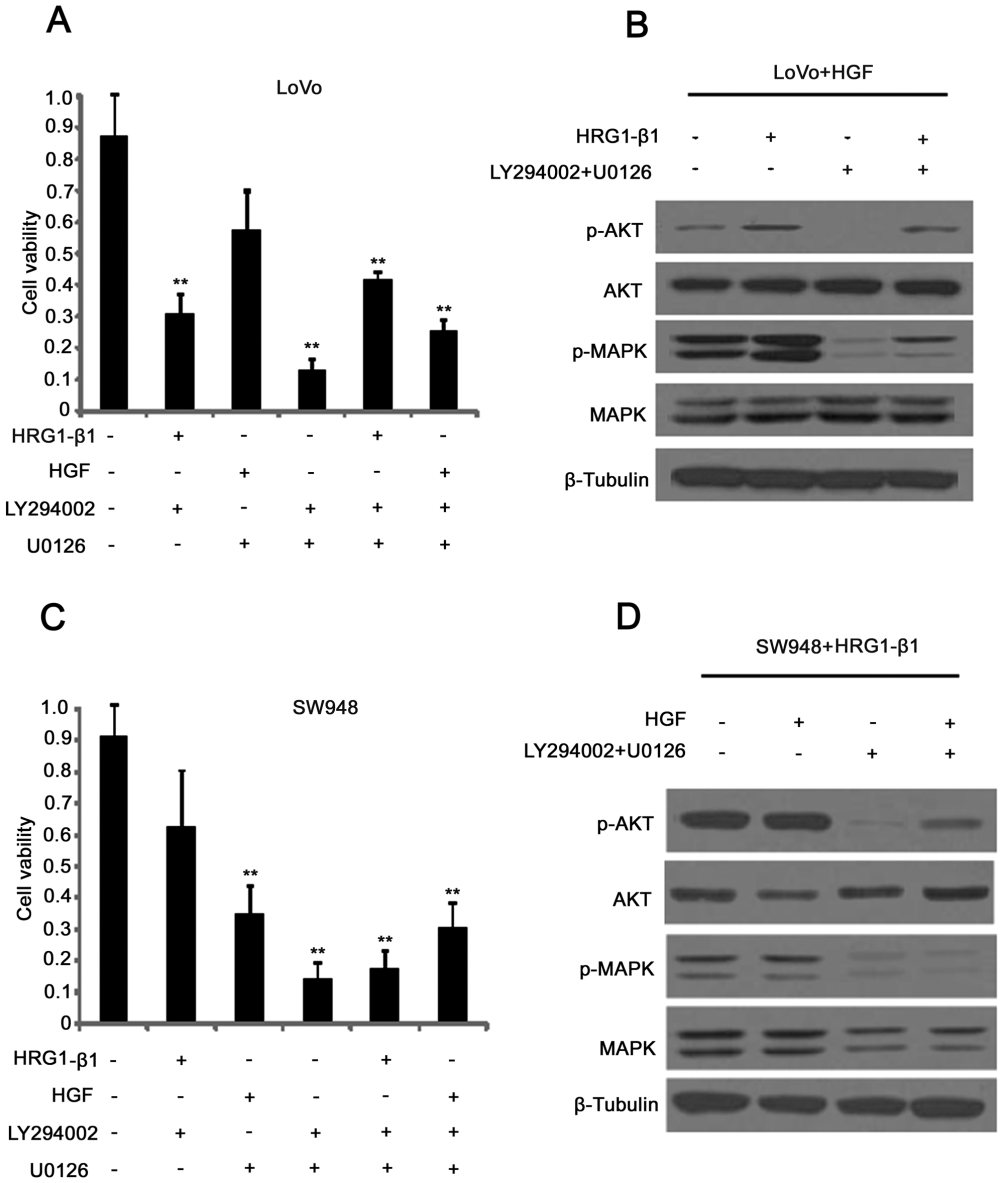
AKT and MAPK are the two cell signaling pathways governing viability of colorectal cancer cell lines with different receptor expression patterns. Differences in cell viability in LoVo (A, B) and SW948 (C, D) cells following treatment with different agonists or inhibitors, as indicated. The data are presented as mean±SD, and **P<0.01 by unpaired Student’s t test.

In order to further investigate the RTK expression variation in colorectal cell lines, a series of tests of cell viability and cell signaling pathways were performed using specific inhibitors of AKT and MAPK. For LoVo cells, cell viability was significantly decreased when treated with LY294002, an inhibitor of AKT signaling. However, there was no significant effect when it was treated with an inhibitor of c-MET, U0126. If LoVo was treated with both inhibitors, the rescue effect of HRG1-β1 on cells was much stronger than that of HGF **(** **Figure 5A** **)**, and signaling activation analysis by western blot also implied that activation of both AKT and MAPK was partially restored by HRG1-β1 even when treated with inhibitors **(** **Figure 5B** **).** This result indicated that cell viability was not very dependent on ErbB3/c-MET/MAPK signaling but instead depended on ErbB3/ErbB2/AKT signaling. However, the situation is not the same in SW948 cells, in which the impairment of cell viability by the inhibitor of c-MET, U0126, was stronger than that caused by LY294002, and the restorative effect was different from that in LoVo cells when treated with both LY294002 and U0126, with the restorative effect of HGF being stronger than HRG1-β1, and the activation of both AKT and MAPK signaling being more strongly induced by HGF than HRG1-β1 **(** **Figure 5C,D** **)**. Taken together, our results show that colorectal cancer exhibits variation in oncogenic RTK due to different molecular patterns of expression including ErbB2/ErbB3/AKT and ErbB3/c-MET/MAPK.

## Discussion

Activation of RTKs is critical for the proliferation and survival of normal and malignant cells. Recently, various RTK activating mutations have been identified in a subset of cancer patients by next generation sequencing, suggesting that constitutive activation of RTKs is one of the underpinning mechanisms of cancer development. However, the remaining population of cancer patients possess no such mutations implying that alternative RTK activating mechanisms exist. Here we show one of such alternative mechanisms is the overexpression of the RTK family members of c-MET, and ErbB2, ErbB3, in CRC cells as well as representative CRC cell lines.

C-MET is one of the receptor tyrosine kinases (RTK) which are widely distributed on the surface of epithelial–endothelial originating cells. Its only known ligand, hepatocyte growth factor (HGF), is expressed mainly in cells of mesenchymal origin [11]. C-MET dimerizes and autophosphorylates upon ligand binding, which in turn creates active docking sites for proteins that mediate serial downstream signaling activation including MAPK, PI3K-AKT, SRC and STAT. Numerous studies have reported that c-MET is overexpressed in a variety of carcinomas including lung, breast, ovary, kidney, colorectal, thyroid, liver, and gastric carcinomas [12]–[14]. It is well known that c-MET plays important roles in cancer development through its proliferation- and angiogenesis-promoting effects exerted by downstream oncogenic pathways, and metastasis-promoting effect which is mainly dependent on metalloprotease production. Due to its role in oncogenesis and cancer progression, c-MET is considered to be an important target in anticancer therapy, and some biological antagonists or monoclonal antibodies targeting c-MET have emerged which are known as c-MET TKI [15]–[18]. Certain gastric cancer and NSCLC cell lines display exquisite sensitivity c-MET TKI, but, cells/tumors treated with c-MET TKI eventually develop resistance. This resistance to c-MET TKI is a result of overcoming the inhibition through sustained high MAPK and PI3K/AKT activity, and EGFR family members can act as complementary receptors for MAPK and PI3K/AKT activation while c-MET is blocked[19]–[22]. While acquisition of novel mutations may be one of the possible explanations to this resistance in clinical treatment, activating mutations of several RTKs including ErbB2, ErbB3 and c-MET were discovered in human colorectal cancer and other cancers [23]–[27]. RTK overexpression and dowmstream signal activation provide an alternative and complimentary mechanism. However, the variability of oncogenic RTK expression levels may also underscore the significance of individualized cancer therapy.

In the present study, we not only confirmed the over-expression of c-MET, ErbB2 and ErbB3 in human colorectal cancer, but also revealed the variation and complementary of these receptors. Moreover, we identified *in vitro* cell line models with different molecular expression patterns which show differing sensitivity to the treatment of both antagonists and agonists of c-MET and EGFR signaling.

The ErbB2 gene is also commonly referred to as Her-2/neu, one of the growth factor receptors leading cell signaling related to cell proliferation, adhesion and movement [28], [29]. It is also a hot target for signaling-based drug development. Trastuzumab, one of the related drugs targeting ErbB2, binds ErbB2 and induces uncoupling of ligand-independent HER2–HER3 heterodimers, thus inhibiting downstream signaling. However, several studies have revealed that trastuzumab treatment can induce resistance both by amplification of signaling through other ErbB receptors and by cross-talk with heterologous RTKs [30]–[32]. As we report in the present study, c-MET has also been found to be upregulated in trastuzumab-resistant ErbB2-overexpressing cells following exposure to trastuzumab. Furthermore, a mechanistic study revealed that activation of c-MET protects cells against trastuzumab by abrogating the induction of p27. This is consistent with the outcome obtained in our study, showing that different molecular patterns exist in human colorectal cancer. Heterogeneous expression of ErbB2 and ErbB3 with varying combinations of RTKs implied potentially different chemotherapy projects.

It is anticipated that an increasing number of molecular signaling pathway-based drugs will emerge, aimed at a variety of targets; however, it is very possible that none of these drugs alone will have a dominant anti-tumor effect. Combination therapy for cancer is an on-going and developing concept and importantly, personal detection and analysis of an individual’s molecular expression pattern is indispensable for more effective therapy with fewer side effects.
